# On a Substitute for McMunn’s Elixir of Opium

**Published:** 1851-08

**Authors:** Eugene Dupuy

**Affiliations:** Pharmaceutist, New York


*On a Substitute for McMunn's Elixir of Opium.* By EUGENE DUPUY, Pharmaceutist, New York.—Within a few years the use of this preparation of opium has been much extended in the United States, through the medium of the press, as well as from the commendation of a numerous class of our practitioners, who found it to possess a sedative property which the ordinary Tincture of Opium does not possess in a similar way. Yet many amongst them reluctantly made use of it, from the fact that its mode of preparation was kept from the public, and that the usual abuse of such preparations, fostered by *directions for use without need of medical aid*, by mothers, nurses, etc., was a great objection to its employment by that class of practitioners who want to *know*, *not only* what is the effect of the medicines they administer, but also, what are their component parts, and how they are prepared. Having such men among the physicians honoring my establishment with their custom, I have endeavored to prepare for their use, substitutes for some of the nostrums possessed of some efficacy. As a result of my endeavors, I will state that my substitute for McMunn's Elixir has been tested for about six years, and that it has been found to possess the sedative property peculiar to it, without any of the unpleasant effects attributed to Laudanum.

The late Dr. Smyth Rodgers, formerly Professor in the New York College of Pharmacy, during his painful illness, had frequent recourse to it, and even preferred it to McMunn's preparation, according to his attending physician's statements, although he had, at first, great reluctance to try anything else. An advantage in my substitute is, that its manipulation is exceedingly simple, and that a country physician having at hand the necessary ingredients, can prepare it as well as the more expert pharmaceutist. I prepare it as follows:

Opium, - - - ʒx

Water, - - - q. s.

Alcohol, 95 p. ct. ℥iv.

The opium is to be made into a thin pulp with water; the mixture allowed to stand in a cool place 48 hours, then transferred into an elongated glass funnel containing filtering paper; a superstratum of water equivalent to the bulk of the whole mass is added. When 12 ounces of liquid have been filtered, the alcohol is added to the filtered solution.*

About two-thirds of the substance of the opium is contained in the solution. The residue, consisting chiefly in resin, caoutchouc and narcotina, together with the ligneous matter. Consequently my substitute is nothing more or less than an aqueous solution of opium, nearly free from narcotina, preserved by alcohol.—*American Journal of Pharmacy.*

*The proportion of opium is the same as that in Tinct. Opii of the U. S. P.

